# A Novel Fat-Augmented Omentum-Based Construct Is a Cost-Effective Alternative for Autologous Breast Reconstruction

**DOI:** 10.3390/jcm14051706

**Published:** 2025-03-03

**Authors:** Pooja S. Yesantharao, Kassandra Carrion, Dung H. Nguyen

**Affiliations:** Division of Plastic & Reconstructive Surgery, Stanford University School of Medicine, Stanford, CA 94304, USA; pyrao@stanford.edu (P.S.Y.); kcarrion@stanford.edu (K.C.)

**Keywords:** breast reconstruction, omental flap, cost effectiveness, Markov model

## Abstract

**Background/Objectives**: The omental fat-augmented free flap (O-FAFF) is a novel technique for autologous breast reconstruction in patients who cannot use or who elect not to use more traditional donor sites. While the clinical outcomes of O-FAFF have been well studied, associated costs and resource utilization have not yet been investigated. The O-FAFF technique involves the use of an acellular dermal matrix and a two-team approach for laparoscopic harvest of the omentum, thereby increasing surgical and materials costs. This study compares the longitudinal cost-effectiveness study of O-FAFF breast reconstruction compared to reconstruction using implants or abdominal donor sites (deep inferior epigastric artery flap or transverse rectus abdominis myocutaneous flap). **Methods**: This cost-effectiveness analysis compared O-FAFF to abdominal free flap and implant-based reconstruction in adults. Markov cohort modeling was used to study cost-effectiveness from the payer perspective. **Results**: Compared to implant-based reconstruction, the incremental cost of O-FAFF reconstruction was USD 9227 and the incremental gain in breast quality-adjusted life-year (B-QALY) was 0.95, resulting in an incremental cost-effectiveness ratio of USD 9712.64/B-QALY gained, which is well under the acceptable cost-effectiveness threshold of USD 50,000 per B-QALY. Compared to abdominal flap reconstruction, O-FAFF reconstruction was associated with an incremental decrease in direct costs of USD 1410.10 and an incremental gain in B-QALYs of 0.36 and was thus the dominant strategy. **Conclusions**: The O-FAFF breast reconstruction technique is a cost-effective alternative to more traditional methods of breast reconstruction, including abdominal free flap techniques and implant-based reconstruction. As such, the O-FAFF technique represents an important novel modality for primary autologous reconstruction.

## 1. Introduction

The omental fat-augmented free flap (O-FAFF) has emerged as a novel technique to provide autologous breast reconstruction for women who cannot use or who elect not to use more traditional donor sites. This flap involves the creation of a “biologic” breast implant using laparoscopically harvested omental tissue encased in acellular dermal matrix (ADM). The ADM is shaped around a breast sizer and is used to provide structural support for the omentum, to create an aesthetic breast shape. Omental tissue volume is augmented with fat grafting as needed to achieve desired breast volumes. The entire construct is then inset in the breast following microanastomosis of the gastroepiploic artery to recipient chest vessels (e.g., internal mammary vessels) [1].

O-FAFF-based breast reconstruction has rapidly gained popularity at our institution and beyond as a safe and clinically efficacious reconstructive modality for patients seeking autologous or hybrid breast reconstruction. Furthermore, longitudinal investigation of O-FAFF patients has demonstrated the durability of this reconstructive technique, with excellent volume retention over time. Ultimately, O-FAFF reconstruction has allowed patients who would not traditionally be candidates for autologous reconstruction (e.g., breast cancer patients with low body mass index) to achieve natural and aesthetic results without breast implants, thereby expanding indications for autologous reconstruction [2]. It is important to note, however, that the O-FAFF technique hinges on the use of ADM and involves laparoscopic harvest of the omentum, which can increase operative costs and operative time, respectively. While the clinical outcomes of the O-FAFF procedure have been well studied [1,2,3], such costs and resource utilization associated with this technique have not yet been formally investigated.

In an increasingly cost-conscious healthcare environment, it is important to consider the costs of medical innovation in addition to clinical outcomes. The O-FAFF technique has demonstrated clinical success, but its cost-efficacy in comparison to more traditional flaps for autologous breast reconstruction (e.g., abdominally based free flaps) or compared to implant-based reconstruction has not been formally characterized. Prior literature has demonstrated that abdominal flap reconstruction is cost effective compared to implants; we aim to investigate whether this association holds for O-FAFF reconstruction [4]. As such, this is a study comparing the longitudinal cost-effectiveness study of O-FAFF breast reconstruction compared to reconstruction using implants or abdominal donor sites (deep inferior epigastric artery flap or transverse rectus abdominis myocutaneous flap).

## 2. Materials and Methods

### 2.1. Model Development

This was a cost-effectiveness analysis comparing O-FAFF to abdominal free flap reconstruction and implant-based reconstruction in adults (≥18 years) who underwent mastectomy for breast cancer. All patients underwent staged breast reconstruction with immediate tissue expander placement at the time of mastectomy and delayed flap or implant reconstruction. Hybrid Monte Carlo patient simulation [5] and Markov cohort modeling [6] were used to study cost-effectiveness from the payer perspective. All analyses were completed in accordance with the Second Panel on Cost-Effectiveness in Health and Medicine using TreeAge Software, 2021 (TreeAge Pro Healthcare Software LLC, Washington, DC, USA).

### 2.2. Data Sources

Study data were derived from institutional data as well as published clinical investigations of staged abdominal flap, implant [7,8] and O-FAFF reconstruction amongst breast patients who underwent mastectomy. Published studies [9,10] and institutional data on quality-of-life outcomes using the BREAST-Q tool were used to measure the effectiveness of each intervention, as is standard in the plastic surgery cost-effectiveness literature. Only studies published within the past 10 years (2014–2024) with at least a level of evidence of III were considered for inclusion, with preference for the highest level of data available (randomized controlled trial, meta-analysis).

### 2.3. Model Parameters

As mentioned above, the model was populated using data derived from institutional data and the published literature. Costs and utilities assigned to each post-operative category are reported in Table 1 and Table 2. An annual discount rate of 3% was used in this model, as is standard in the literature [11,12].

Direct costs evaluated in this study included surgical costs (both physician and facility fees) and utilization of acute medical services for postoperative care (3 days postoperative inpatient stay for flap reconstruction versus outpatient surgery for implant reconstruction [10]), as well as pain medication costs after surgery. Direct costs were determined using Medicare reimbursement fees. As all included patients were assumed to have undergone staged reconstruction with immediate prepectoral tissue expander placement after surgery, the costs of the initial mastectomy/expander placement and preoperative visits for tissue expander fills were assumed to be the same for all treatment arms and were thus not included in study calculations. The cost of acellular dermal matrix was included for O-FAFF and implant-based breast reconstruction, but not for abdominal flap reconstruction. The costs of inpatient stay were included for abdominal flap reconstruction and O-FAFF reconstruction.

All model costs were inflated to 2024 US dollars. For the lifetime horizon, mortality was determined using age- and sex-specific all-cause mortality rates calculated from the Center for Disease Control and Prevention National Vital Statistics Report, adjusted for the additional risk of undergoing general anesthesia for an ambulatory procedure in patients with a comorbid diagnosis of breast cancer.

The unit of measure for effectiveness was Breast Quality Adjusted Life Years (B-QALYs), as is standard in the breast reconstruction cost-effectiveness literature. B-QALYs were deducted from patients who experienced postoperative complications, based on established methods from the literature [13,14] and review of the institutional data regarding changes in BREAST-Q scores amongst patients experiencing postoperative complications. Those who underwent elective postoperative revision surgery did not achieve maximal post-reconstruction B-QALYs until after their revision procedure, in accordance with the published literature on quality-of-life outcomes amongst patients undergoing revision surgery after breast reconstruction [15,16,17].

### 2.4. Model Assumptions

To simplify the Markov model, the patients entering the model in each cohort were standardized, such that calculations were based on assumptions that patient characteristics such as average body mass index, history of radiation therapy, smoking status, diabetes, etc., were equal amongst all three reconstructive cohorts. Furthermore, any patient entering the base case model was assumed to have been an appropriate candidate for any of the three reconstructive procedures (i.e., implant reconstruction, abdominal flap reconstruction, and O-FAFF reconstruction). Only pre-pectoral tissue expander and implant placement was considered, as this is the dominant implant-based reconstructive strategy used at our institution.

The model was run comparing both implant reconstruction with O-FAFF reconstruction and abdominal flap reconstruction with O-FAFF reconstruction to account for both patients who may be candidates for all three procedures as mentioned above, as well as for patients who may not be candidates for more traditional abdominal donor sites (e.g., low body mass index, prior extensive abdominal surgery or prior abdominoplasty, etc.).

### 2.5. Patient Simulation

A cohort of 10,000 patients entered the model one at a time using Monte Carlo microsimulation trials and received either O-FAFF, implant, or abdominal free flap breast reconstruction. In the base case, the model time horizon was 2 years with cycles of 1 month each, given that available institutional data largely spans a 2-year postoperative period. Upon entering the model, patients cycled through several postoperative states based on pooled complication/failure and revision surgery rates for each reconstruction type, as derived from our institutional sample and the available literature. All patients were followed in the model until the earliest of two outcomes: end of the time horizon or death.

### 2.6. Study Outcomes

The primary model outcome was the incremental cost-effectiveness ratio (ICER), which is represented in terms of cost per breast quality-adjusted life year (B-QALY) gained. In the base case, outcomes were analyzed over a 5-year horizon with monthly cycles, as this is the longest available data metric for the O-FAFF cohort given its relative novelty. Aggregated costs/utilities throughout the entire model time horizon were used to determine the ICER for each treatment. ICERs were determined by dividing the difference in cost between O-FAFF, implant and abdominal flap reconstruction by the difference in B-QALYs between the treatment arms. The willingness-to-pay threshold was set at USD 50,000 per B-QALY, as is standard in the plastic surgery cost-effectiveness literature.

### 2.7. Sensitivity Analyses

One-way deterministic sensitivity analyses were used to evaluate model robustness. Parameters were varied ±15% from model base case values. Probabilistic sensitivity analyses were also conducted by using second-order Monte Carlo simulation drawing from preset distribution functions. Costs were varied following a gamma distribution, and proportions were varied following a beta distribution. In total, five thousand model simulations were run.

## 3. Results

The pooled age and clinical characteristics of study patients were tabulated using institutional data and the literature. The values described relate to the existing population of O-FAFF patients at this institution. Mean age was 52 years (standard deviation 9 years) and mean body mass index was 22 (standard deviation 3). See Table 3 for the demographic and clinical characteristics of each study cohort, organized according to flap (O-FAFF, abdominal free flap reconstruction, and implant reconstruction).

### 3.1. Model Outcomes

**Figure 1:**
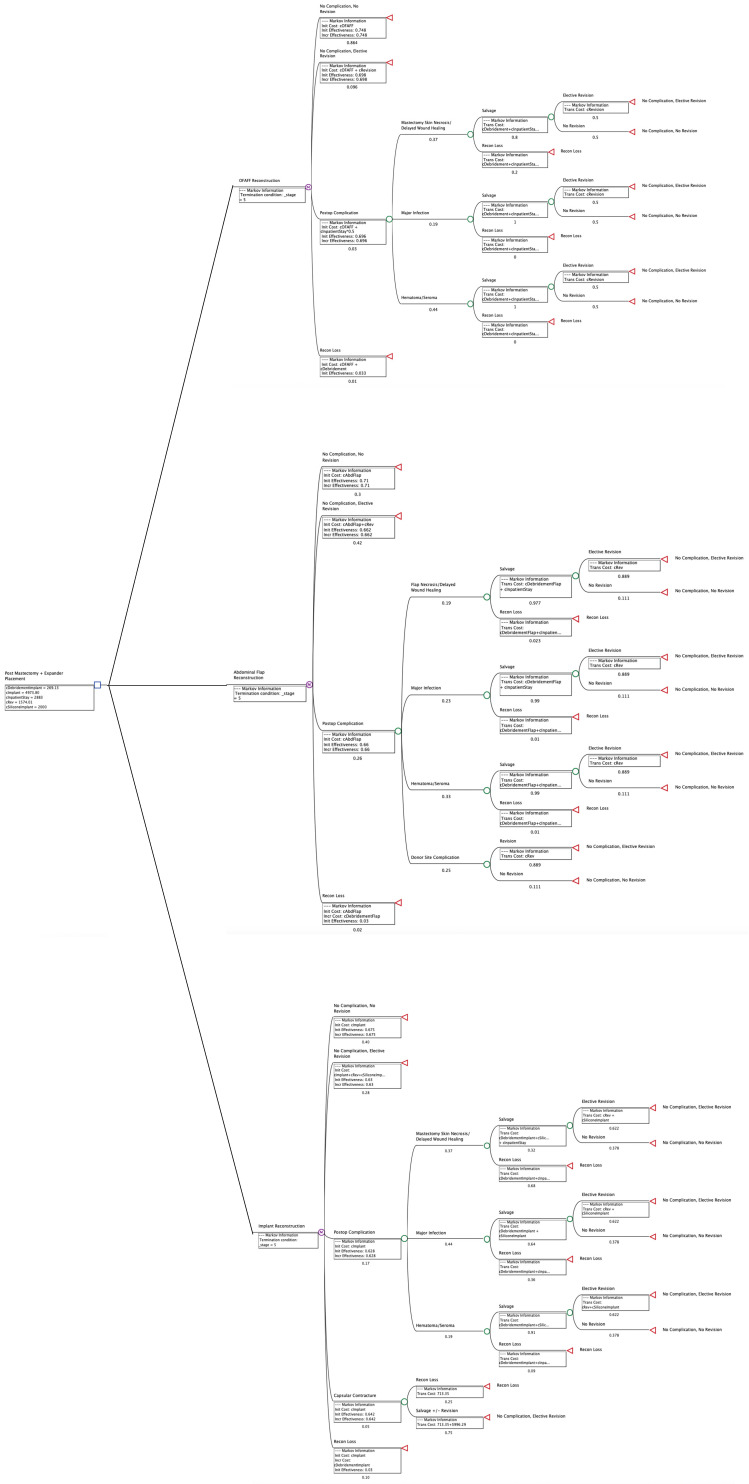
Breast Reconstruction Cohort Model.

Model results were generated from the payer perspective, using US dollars. Model calculations were run over the 5-year time horizon, as mentioned above (Table 4; Figure 1). O-FAFF was compared to other subgroups from Table 3—Abdominal Flap and Implant.

Compared to implant-based reconstruction, the incremental cost of O-FAFF reconstruction was USD 9227 and the incremental gain in B-QALY was 0.95. This translated to an incremental cost-effectiveness ratio of USD 9712.64/B-QALY gained, which is well under the acceptable cost-effectiveness threshold of USD 50,000 per B-QALY.

Compared to abdominal flap reconstruction, O-FAFF reconstruction was associated with an incremental decrease in direct costs of USD 1410.10 and an incremental gain in B-QALYs of 0.36. As such, O-FAFF was found to be the dominant strategy compared to abdominal flap reconstruction, given the increase in effectiveness combined with the decrease in cost.

### 3.2. Sensitivity Analyses

#### 3.2.1. Deterministic Sensitivity

Table 5 demonstrates the results of multiple rounds of one-way deterministic sensitivity analyses. Overall, O-FAFF remained cost effective despite variation across the model inputs tested. When the model was run over to a lifetime horizon, the aforementioned model findings remained unchanged.

#### 3.2.2. Probabilistic Sensitivity Analyses

**Figure 2:**
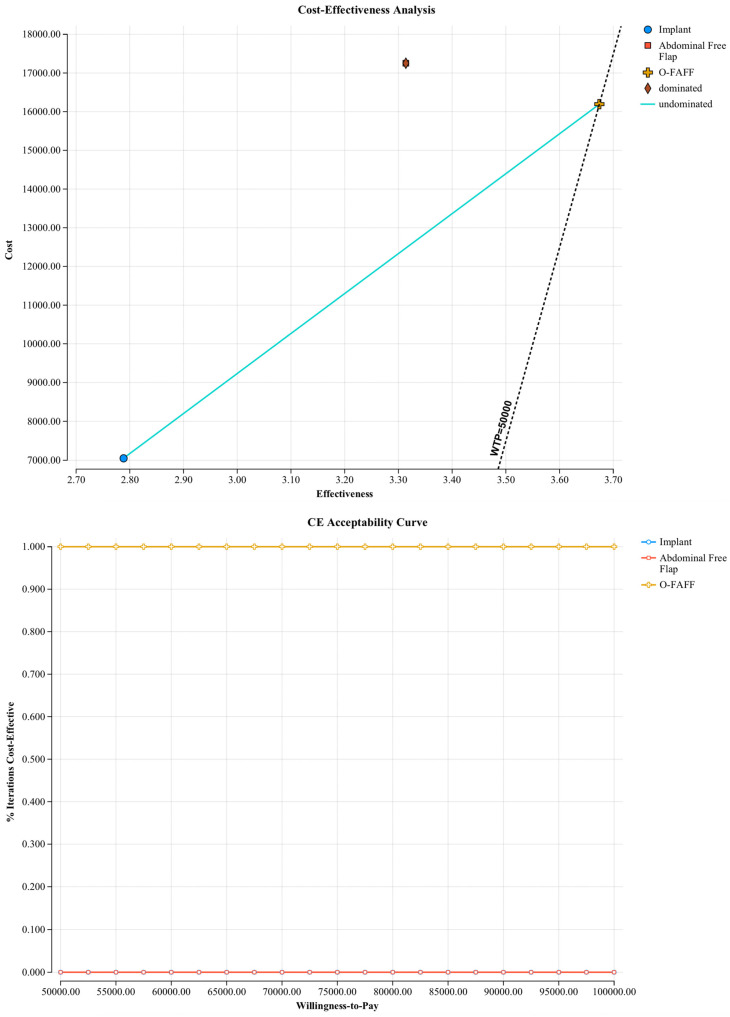

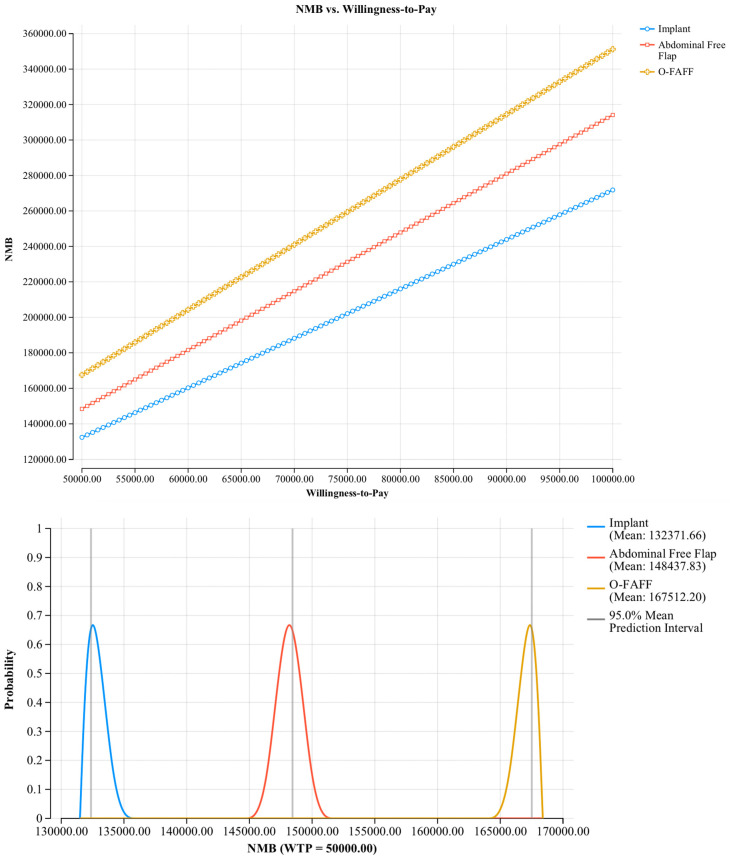
Cost–utility acceptability curves.

**Figure 3:**
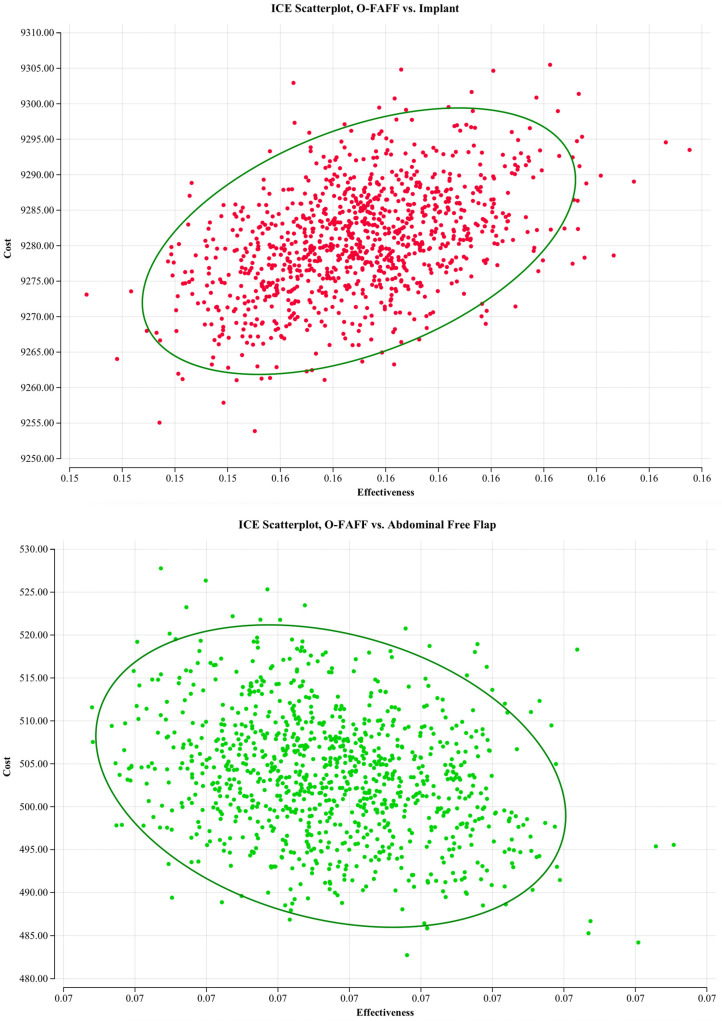
Incremental costs plot.

Probabilistic sensitivity analyses were undertaken to evaluate the model against multiple willingness-to-pay thresholds (USD 50,000; USD 100,000; and USD 200,000 per B-QALY). Figure 2 demonstrates cost–utility acceptability curves, displaying the probability that O-FAFF reconstruction is cost-effective when compared to these willingness-to-pay thresholds. Figure 3 is a scatterplot demonstrating incremental costs versus incremental B-QALYs. O-FAFF reconstruction remained cost-effective in 99% of the simulations run.

## 4. Discussion

The O-FAFF surgical technique represents a paradigm shift in breast reconstruction, by re-framing the omental flap as a primary means to achieve an aesthetic reconstructed breast rather than as a salvage procedure. Furthermore, the O-FAFF technique has expanded indications for autologous reconstruction, by allowing patients who cannot use more traditional donor sites (e.g., those with low body mass index) to achieve natural-appearing reconstructed breasts using their own tissue [2]. However, this technique is more expensive up front than abdominal free flap and implant-based reconstruction as it involves the use of the acellular dermal matrix and laparoscopic harvest of the omentum, which increase surgical costs and operative time, respectively. However, we hypothesized that the initial costs of the O-FAFF technique may be offset by longitudinal improvements in pain, postoperative recovery, and patient satisfaction, resulting in shorter hospitalizations and fewer downstream revision surgeries. In fact, we have previously demonstrated the clinical safety of the O-FAFF and the longitudinal durability of its results [3]. The current study builds on our prior work to investigate the relative cost-efficacy of the O-FAFF in comparison to abdominal free flap reconstruction and implant-based reconstruction over a five-year time horizon, to present a comprehensive analysis of this novel breast reconstruction technique.

### 4.1. Model Outcomes

In our study, we demonstrate that the O-FAFF technique is cost-effective compared to both implant-based and abdominal flap reconstruction. As expected, the O-FAFF technique was more expensive than implant-based reconstruction, with a similar incremental increase in direct costs compared to reconstructive implants, as has been demonstrated in the cost-effectiveness literature comparing abdominal flaps to implant reconstruction [18]. However, when factoring in the gain in breast quality adjusted life years, the incremental cost-effectiveness ratio was well below the standard willingness-to-pay threshold, suggesting that O-FAFF is cost-effective when compared to implant-based reconstruction. When compared to abdominal flaps, O-FAFF was found to be the dominant strategy, resulting in both decreased cost and increased effectiveness. The omentum is a highly biologically active donor site with both immunologic and physiologic functionality beyond simply providing soft tissue filler. As such, decreased costs in comparison to abdominal flap reconstruction [19] were noted to be a result of decreased postoperative complication rates (e.g., decreased severe infections). Furthermore, the O-FAFF technique was also associated with lower downstream revision rates. This may be due to a combination of reduced donor site morbidity and the rich vascular network of the omentum, decreasing the risk of complications such as fat necrosis and promoting retention of engrafted fat to provide stable breast volume over time. Taken together, these findings likely contributed to fewer elective revisions in O-FAFF patients compared to abdominal flap patients, thereby decreasing longitudinal costs.

While our model investigated direct costs of breast reconstruction, it is also important to consider the indirect costs of each reconstructive technique (i.e., productivity loss). While autologous reconstruction has been repeatedly demonstrated to improve longitudinal quality of life outcomes [20], one drawback of this technique compared to implant-based reconstruction is the associated donor site morbidity. Donor site morbidity can result in increased immediate postoperative pain, decreased postoperative mobility, longer hospitalization, and a slower return to work [21]. In the long run, donor site morbidity can result in the need for revision surgery to address hernias, abdominal wall laxity, or adverse scarring. All of the aforementioned complications can increase time to return to baseline activities and work, thereby increasing indirect costs [22]. The O-FAFF technique utilizes laparoscopic omental harvest to minimize donor site morbidity, which has been shown to decrease postoperative pain and hospitalization. Furthermore, no O-FAFF patients have needed downstream donor site revision surgery, which reduces time off work for revision surgery or due to donor site symptoms. Ultimately, the O-FAFF technique combines the benefits of autologous reconstruction with greatly reduced donor site morbidity. As such, the O-FAFF technique can decrease indirect costs, thereby suggesting the additional cost-efficacy of this technique from the societal perspective compared to implant-based reconstruction and traditional abdominal flaps.

### 4.2. Study Limitations

As always, this study is not without limitation. First, model parameters were populated by using available clinical data from our institution and from the literature. Although only well-designed clinical studies with a level of evidence of at least III were selected, the relative novelty of the O-FAFF technique has limited the type of data available for this modality. As such, O-FAFF model parameters were not based on as robust of a dataset as the other two study arms (abdominal free flap reconstruction and implant-based reconstruction), and multiple assumptions had to be made regarding longitudinal treatment effects and costs for the O-FAFF cohort when the model horizon was extended beyond 5 years for sensitivity analyses. Furthermore, there are limitations to the study analyses that are inherent to models based on assumptions. For instance, as in prior breast reconstruction cost-effectiveness analyses, patients could only experience one complication at a time when cycling through Markov states, which does not exactly reflect what may happen clinically (e.g., a patient can concurrently have an infection and mastectomy skin necrosis, which can be addressed in one combined debridement procedure). However, this is a limitation of most available cost-effectiveness models in the breast reconstruction literature, and this model logic was common across all three treatment cohorts (implant, abdominal flap, and O-FAFF reconstruction) in the constructed model.

### 4.3. Future Directions

Breast reconstruction is a continually evolving field, and assessing the cost-effectiveness of novel methods such as the O-FAFF provides important insight into their relative utility in the growing armamentarium of breast reconstruction techniques currently available. Breast reconstruction is undertaken for the primary purpose of improving quality of life following mastectomy for breast cancer, and as such including the effectiveness component in terms of B-QALYs is paramount in such cost analyses. Particularly in the low body mass index population, autologous reconstruction options are limited and often implants are often the only form of reconstruction offered. However, the literature has overwhelmingly demonstrated the superior quality of life outcomes with autologous reconstruction versus breast implants, especially longitudinally over time. Furthermore, O-FAFF reconstruction holds several other benefits, including lower donor site morbidity, less postoperative pain, and shorter hospitalizations, all of which can improve quality of life. In combination with longitudinal cost savings as a result of reduced short-term complications and fewer long-term revision surgeries, the O-FAFF represents a cost-effective technique for breast reconstruction. Further work will include prospective, multi-center work to better characterize the longitudinal outcomes and relative utility of O-FAFF breast reconstruction.

## 5. Conclusions

The O-FAFF breast reconstruction technique was demonstrated to be a cost-effective alternative to more traditional methods of breast reconstruction, including abdominal free flap techniques and implant-based reconstruction. Compared to breast implants, O-FAFF reconstruction was found to increase costs but also increase effectiveness, resulting in an incremental cost-effectiveness ratio well below the willingness-to-pay threshold. Compared to abdominal flaps, O-FAFF represented the dominant strategy due to reduced complication rates and revision needs over time, as well as decreased postoperative pain and hospitalization. These findings were stable on both probabilistic and deterministic sensitivity analyses. This suggests that O-FAFF is a cost-effective breast reconstruction technique both for patients who are candidates for all three procedures and for those who are not candidates for abdominal flaps. As such, the O-FAFF technique represents an important step forward in breast reconstruction, and continued work is needed to investigate this technique as it gains traction as a novel modality for primary autologous reconstruction.

## Figures and Tables

**Table 1:** Model cost parameters (2024, USD), stratified by cohort.

Direct Costs	O-FAFF	Abdominal Flap	Implant	References
Surgical + Materials Cost	USD 7974.48	USD 2703.94	USD 4973.82	Institutional Data, Center for Medicare and Medicaid Services
Anesthesia Cost	USD 828.29	USD 580.35	USD 197.54	
Hospital Stay	USD 8649.00	USD 8649.00	USD 0	
Reconstructive Salvage	USD 350.87	USD 3205.04	USD 2269.13	
Reconstructive Loss	USD 350.87	USD 350.87	USD 269.13	
Revision Surgery	USD 1574.01	USD 1574.01	USD 1574.01	

**Table 2:** Model utility parameters (B-QALY), stratified by cohort.

	Expected B-QALYs			References
	O-FAFF	Abdominal Flap	Implant	
No Complications	3.74	3.55	3.38	Institutional Data
Reconstructive Loss	0.03	0.03	0.08	
Reconstructive Salvage	3.48	3.30	3.14	
Revision Surgery	3.49	3.31	3.15	

**Table 3:** Clinical and demographic model parameters, stratified by treatment.

	O-FAFF	Abdominal Flap	Implant	References
Age—years (SD)	49 (8)	51 (9)	55 (9)	Institutional Data
BMI—mean (SD)	22.1 (3)	27.4 (5)	26.1 (4)	
Nipple Sparing Mastectomy	78%	35%	59%	
Follow Up—months (SD)	22(8)	49(10)	72(12)	

**Table 4:** Model results.

	Payer Perspective
O-FAFF versus Implant	
Incremental costs per patient	+USD 9227
Incremental QALYs per patient	+0.95
ICER	USD 9712.64
O-FAFF versus Abdominal Flap	
Incremental costs per patient	−USD 1410.10
Incremental QALYs per patient	+0.36
ICER	Dominant

**Table 5:** One-way deterministic sensitivity analyses.

	Payer Perspective		
	Low	Base	High
O-FAFF versus Implant			
Surgical Costs	USD 6201.39	USD 8024.12	USD 12,102.20
Time Horizon	USD 7231.04	USD 9584.39	USD 10,390.10
O-FAFF versus Abdominal Flap			
Surgical Costs	D	D	D
Time Horizon	D	D	D

D: O-FAFF is dominant strategy.

## Data Availability

Data are unavailable due to privacy or ethical restrictions.

## Abbreviations

The following abbreviations are used in this manuscript:

USD	United States Dollar
OR	Operating Room

## References

[1] Nguyen D.H., Ma I.T., Choi Y.K., Zak Y., Dua M.M., Wapnir I.L. (2022). Creating a Biological Breast Implant with an Omental Fat-Augmented Free Flap. Plast. Reconstr. Surg..

[2] Devisetti N., Sarpong C., Hu A.C., Yesantharao P.S., Liu F.C., Carrion K., Nguyen D.H. (2024). Fat-augmented omentum-based construct for breast reconstruction. Plast. Aesthetic Res..

[3] Nguyen D.H., Rochlin D.H., Deptula P.L., Zak Y., Dua M., Wapnir I.L. (2022). A Novel Fat-Augmented Omentum-Based Construct for Unilateral and Bilateral Free-Flap Breast Reconstruction in Underweight and Normal Weight Women Receiving Nipple or Skin-Sparing Mastectomies. Ann. Surg. Oncol..

[4] Matros E., Albornoz C.R., Razdan S.N., Mehrara B.J., Macadam S.A., Ro T., McCarthy C.M., Disa J.J., Cordeiro P.G., Pusic A.L. (2015). Cost-Effectiveness Analysis of Implants versus Autologous Perforator Flaps Using the BREAST-Q. Plast. Reconstr. Surg..

[5] Duane S., Kennedy A., Pendleton B.J., Roweth D. (1987). Hybrid Monte Carlo. Phys. Lett. B.

[6] Iskandar R., Berns C. (2022). Markov Cohort State-Transition Model: A Multinomial Distribution Representation. Med. Decis. Mak..

[7] Eltahir Y., Werners L.L.C.H., Dreise M.M., van Emmichoven I.A.Z., Werker P.M.N., de Bock G.H. (2015). Which Breast Is the Best? Successful Autologous or Alloplastic Breast Reconstruction. Plast. Reconstr. Surg..

[8] Blok Y.L.M., van Lierop E., Plat V.D., Corion L.U., Verduijn P.S., Krekel N.M. (2021). Implant Loss and Associated Risk Factors following Implant-based Breast Reconstructions. Plast. Reconstr. Surg. Glob. Open.

[9] Razdan S.N.M., Cordeiro P.G., Albornoz C.R.M., Ro T.B., Cohen W.A., Mehrara B.J., McCarthy C.M.M., Disa J.J., Pusic A.L., Matros E.M. (2016). Cost-Effectiveness Analysis of Breast Reconstruction Options in the Setting of Postmastectomy Radiotherapy Using the BREAST-Q. Plast. Reconstr. Surg..

[10] Zong A.M., Leibl K.E., Weichman K.E. (2024). Effects of Elective Revision after Breast Reconstruction on Patient-Reported Outcomes. J. Reconstr. Microsurg..

[11] Sussman M., Benner J., Neumann P., Menzin J. (2018). Cost-effectiveness analysis of erenumab for the preventive treatment of episodic and chronic migraine: Results from the US societal and payer perspectives. Cephalalgia.

[12] Payne K.A., Varon S.F., Kawata A.K., Yeomans K., Wilcox T.K., Manack A., Buse D.C., Lipton R.B., Goadsby P.J., Blumenfeld A.M. (2011). The International Burden of Migraine Study (IBMS): Study design, methodology, and baseline cohort characteristics. Cephalalgia.

[13] Liu C., Zhuang Y., Momeni A., Luan J., Chung M.T., Wright E., Lee G.K. (2014). Quality of life and patient satisfaction after microsurgical abdominal flap versus staged expander/implant breast reconstruction: A critical study of unilateral immediate breast reconstruction using patient-reported outcomes instrument BREAST-Q. Breast Cancer Res. Treat..

[14] Pirro O., Mestak O., Vindigni V., Sukop A., Hromadkova V., Nguyenova A., Vitova L., Bassetto F. (2017). Comparison of Patient-reported Outcomes after Implant Versus Autologous Tissue Breast Reconstruction Using the BREAST-Q. Plast. Reconstr. Surg. Glob. Open.

[15] Kandi L.A.B., Hammond J.B., Nadone H.B., Kosiorek H.E., Rebecca A.M.M., Casey W.J.I., Reece E.M.M., Cronin P.A., Pockaj B.A. (2023). Patient Perspectives and Quality of Life after Breast Reconstruction and the Impact of Subsequent Revisions. Plast. Reconstr. Surg. Glob. Open.

[16] Kim M.B., Vingan P.B., Boe L.A., Mehrara B.J., Stern C.S., Allen R.J., Nelson J.A. (2024). Satisfaction with Breasts following Autologous Reconstruction: Assessing Associated Factors and the Impact of Revisions. Plast. Reconstr. Surg..

[17] Nelson J.A., Voineskos S.H., Qi J., Kim H.M., Hamill J.B., Wilkins E.G., Pusic A.L. (2019). Elective Revisions after Breast Reconstruction: Results from the Mastectomy Reconstruction Outcomes Consortium. Plast. Reconstr. Surg..

[18] Asaad M., Slovacek C.B., Mitchell D.B., Liu J., Selber J.C.M., Clemens M.W., Chu C.K.M., Mericli A.F., Butler C.E. (2022). Surgical and Patient-Reported Outcomes of Autologous versus Implant-Based Reconstruction following Infected Breast Device Explantation. Plast. Reconstr. Surg..

[19] Francis S.D.M., Thawanyarat K.B., Johnstone T.M.B., Yesantharao P.S., Kim T.S.B., Rowley M.A.B., Sheckter C.C., Nazerali R.S.M. (2023). How Postoperative Infection Affects Reoperations after Implant-based Breast Reconstruction: A National Claims Analysis of Abandonment of Reconstruction. Plast. Reconstr. Surg. Glob. Open.

[20] Grover R., Padula W.V., Van Vliet M., Ridgway E.B. (2013). Comparing Five Alternative Methods of Breast Reconstruction Surgery. Plast. Reconstr. Surg..

[21] Toyserkani N.M., Jørgensen M.G., Tabatabaeifar S., Damsgaard T., Sørensen J.A. (2020). Autologous versus implant-based breast reconstruction: A systematic review and meta-analysis of Breast-Q patient-reported outcomes. J. Plast. Reconstr. Aesthetic Surg..

[22] Thoma A., Veltri K., Khuthaila D., Rockwell G., Duku E. (2004). Comparison of the Deep Inferior Epigastric Perforator Flap and Free Transverse Rectus Abdominis Myocutaneous Flap in Postmastectomy Reconstruction: A Cost-Effectiveness Analysis. Plast. Reconstr. Surg..

